# The emerging roles of small nucleolar RNAs, piwi-interacting RNAs, tRNA-derived small RNAs, and circular RNAs in the regulation of cardiac hypertrophy, ischemic heart disease, and heart failure

**DOI:** 10.1016/j.gendis.2025.101996

**Published:** 2025-12-19

**Authors:** Jingyi Wang, Jingyi Xie, Hao Chen, Wenxu Wang, Qing Liu, Xuanzhen Guo, Kun Wang, Jie Ju

**Affiliations:** a Morphology Lab, School of Basic Medical Sciences, Shandong Second Medical University, Weifang, Shandong 261053, China; b Neurologic Disorders and Regenerative Repair Laboratory, Shandong Second Medical University, Weifang, Shandong 261053, China; c Department of Physiology, School of Basic Medical Sciences, Shandong Second Medical University, Weifang, Shandong 261053, China; d Institute for Translational Medicine, The Affiliated Hospital of Qingdao University, College of Medicine, Qingdao University, Qingdao, Shandong 266021, China

**Keywords:** Cardiac hypertrophy, Heart failure, Ischemic heart disease, Medical value, ncRNAs

## Abstract

Heart disease, including cardiac hypertrophy, ischemic heart disease, and heart failure, remains a global health challenge. Given the limitations of existing diagnostic and treatment methods, finding new biomarkers and therapies is essential. Non-coding RNAs (ncRNAs) are gaining prominence as key regulators in the medical field, playing a pivotal role in modulating the onset and progression of heart disease. Their significant potential as diagnostic biomarkers and therapeutic targets is increasingly recognized. This article comprehensively analyzes the basic characteristics of small nucleolar RNAs (snoRNAs), piwi-interacting RNAs (piRNAs), tRNA-derived small RNAs (tsRNAs), and circular RNAs (circRNAs), along with their recent research progress in cardiac hypertrophy, ischemic heart disease, and heart failure. By elucidating their functional mechanisms and clinical potential, this review provides foundational insights for translating ncRNA research into medical applications.

## Introduction

Heart disease is the leading cause of death worldwide.^1^ Cardiac hypertrophy, ischemic heart disease (IHD), and heart failure (HF) are common cardiovascular conditions that pose a serious threat to human health or life.^2^, ^3^, ^4^, ^5^ Cardiac hypertrophy, characterized by an increase in left ventricular mass due to thickening of the left ventricular wall and/or ventricular diameter enlargement, manifests in two distinct forms: physiological and pathological hypertrophy. While both types initially serve as adaptive responses, pathological hypertrophy ultimately becomes maladaptive, leading to fibrosis, apoptosis, and structural dysfunction that may progress to HF.^2^ This pathological condition can be triggered by various factors, including obesity, hypertension, and genetic predispositions.^2^ IHD is defined as heart disease resulting from myocardial ischemia, an imbalance between myocardial oxygen supply and demand primarily due to the narrowing or blockage of the coronary arteries.^5^ Its most severe acute manifestation is myocardial infarction (MI), typically caused by an acute thrombotic occlusion of a coronary artery, resulting in irreversible ischemic damage. A key pathological feature of MI is myocardial necrosis, which initiates cardiac remodeling, a maladaptive change that can ultimately culminate in HF.^1^^,^^3^^,^^5^ Patients with IHD often require percutaneous coronary intervention; however, the procedure itself carries the risk of myocardial ischemia/reperfusion (I/R) injury.^3^ HF, representing the terminal common pathway of numerous cardiac diseases, occurs when cardiac output becomes inadequate to meet metabolic demands, with cardiac hypertrophy and IHD being the two major contributing factors.^2^^,^^4^, ^5^, ^6^ With continued innovation in medical technology, there has been significant progress in the prevention, diagnosis, treatment, and prognosis of heart disease, but its risks still cannot be ignored.^1^ It is crucial to further understand the pathogenesis of heart disease and the signaling pathways involved, toward the discovery of novel biomarkers and targetable pathways.^1^^,^^7^

The pathogenesis and progression of heart disease are regulated by diverse molecular mechanisms. In recent years, the role of non-coding RNAs (ncRNAs) in heart disease has gained increasing attention. ncRNAs are primarily categorized by molecular size into two major classes: small non-coding RNAs (sncRNAs, < 200 nucleotides [nt]) and long non-coding RNAs (lncRNAs, > 200 nt). Among these, the sncRNA family encompasses multiple subtypes, including microRNAs (miRNAs), small nucleolar RNAs (snoRNAs), piwi-interacting RNAs (piRNAs), and tRNA-derived small RNAs (tsRNAs).^1^^,^^8^, ^9^, ^10^, ^11^ Additionally, the role of circular RNAs (circRNAs), a novel class of ncRNAs with circular structures, in cardiovascular diseases has been recognized.^12^ As their name implies, ncRNAs are not translated into proteins but serve as crucial regulators of biological processes.^12^ Among ncRNAs, miRNAs and lncRNAs stand as the most comprehensively investigated in cardiovascular disease research. However, emerging research has demonstrated that other ncRNA species play significant roles in heart disease as well and may serve as potential biomarkers and therapeutic targets.^13^

In this review, we focus on the characterization, biosynthesis, and functions of snoRNAs, piRNAs, tsRNAs, and circRNAs, with particular emphasis on their roles in cardiac hypertrophy, IHD, and HF. By synthesizing the latest research advancements in this field, we aim to provide valuable theoretical insights that may advance emerging diagnostic approaches and therapeutic strategies for heart disease.

## snoRNAs

### Classification, biosynthesis, and function of snoRNAs

**Figure 1:**
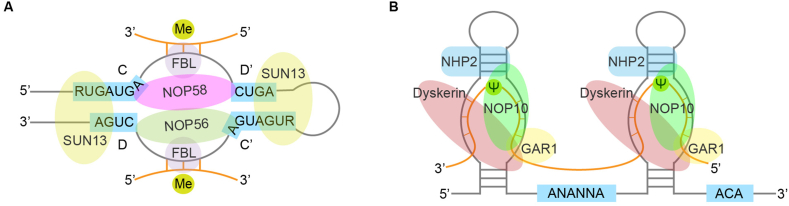
Structural characteristics and binding core proteins of C/D Box and H/ACA Box snoRNAs. **(A)** Structure of C/D Box snoRNA. C/D Box snoRNA contains two conserved motifs (C Box and D Box), two less conserved motifs (C′ Box and D′ Box), a hairpin structure, and a large internal loop that forms a “kink-turn” (K-turn) motif. The K-turn motif serves as the scaffold for core protein binding. The core proteins that bind to C/D Box snoRNA include FBL, NOP58, NOP56, and SNU13. C/D Box snoRNAs direct 2′-O-methylation (2′-O-Me) modification of target RNAs through base pairing, typically occurring in the 5′ upstream region of the D Box and D′ Box. **(B)** Structure of H/ACA Box snoRNA. H/ACA Box snoRNA is characterized by a “hairpin-hinge-hairpin-tail” secondary structure. Two motifs, H Box and ACA Box, are located in the hinge region and the tail region, respectively. The H/ACA Box snoRNA assembles into a ribonucleoprotein (snoRNP) complex through interactions with four core proteins: Dyskerin, NOP10, NHP2, and GAR1. H/ACA Box snoRNA directs pseudouridylation modification on target RNA, with the complementary sequences to the target RNA located in the internal loops of the hairpin. Figure created with Adobe Illustrator.

snoRNAs are abundant, conserved sncRNAs that are primarily localized to the nucleolus with a characteristic size distribution typically spanning 60–300 nt. Based on structural motifs and biological roles, four principal groups exist: C/D Box snoRNAs, H/ACA Box snoRNAs, small Cajal body (CB)-specific RNAs (scaRNAs), and orphan snoRNAs, with C/D Box snoRNAs and H/ACA Box snoRNAs being the dominant types.^1^^,^^5^^,^^14^^,^^15^ Different snoRNAs form ribonucleoprotein (RNP) complexes through interactions with specific core proteins, and their most typical role is to guide modifications by base-pairing with target RNAs.^14^ C/D Box snoRNAs contain two conserved motifs, the C Box (RUGAUGA) and D Box (CUGA), as well as two less conserved motifs, the C′ Box and D′ Box. The C′ Box and D′ Box can form the “kink-turn” (K-turn) motif via non-canonical G-A base pairing, which serves as the scaffold for snoRNP assembly (*e.g.*, FBL, NOP58, NOP56, and SNU13) and is closely related to its biological function (Fig. 1A).^1^^,^^14^^,^^15^ H/ACA Box snoRNAs are characterized by a “hairpin-hinge-hairpin-tail” secondary structure, containing two conserved motifs: the H Box (ANANNA, where N can be any nucleotide) and the ACA Box, located in the hinge and tail regions, respectively. Upstream of the hinge and tail regions, two pseudouridylation loops exhibit sequence complementarity with the target RNA. H/ACA Box snoRNAs can form snoRNPs with Dyskerin, NOP10, NHP2, and GAR1 to participate in target RNA modification (Fig. 1B).^1^^,^^14^^,^^15^ scaRNAs are located in CBs and have structures similar to either C/D Box snoRNAs, H/ACA Box snoRNAs, or both. Furthermore, scaRNAs contain additional motifs: the CAB Box (UGAG) and GU-repeat bound, which are associated with their localization to CBs.^1^^,^^14^ Orphan snoRNAs lack validated modification targets but may have other important functions.^1^^,^^14^

snoRNAs are either transcribed by independent promoters in separate transcription units or encoded by introns without independent promoters.^1^^,^^14^ These two coding organizations are conserved across species but differ in their proportions. For example, in yeast, most snoRNAs are encoded in separate transcription units, while humans mostly harbor them within intronic sequences.^14^

Typical functions of snoRNAs are 2′-O-methylation (2′-O-Me) guided by C/D Box snoRNAs, and pseudouridylation (Ψ) guided by H/ACA Box snoRNAs (Fig. 1A and B). With advances in research, some atypical functions of snoRNAs have been discovered, such as alternative splicing (AS), 18S rRNA acetylation, DNA damage regulation, and the promotion of protein translocation and secretion.^1^^,^^5^^,^^16^ Additionally, the discovery of extracellular snoRNAs implies their potential involvement in signal transduction and cellular communication.^17^

In heart disease, snoRNA research has been mostly focused on omics studies, and differences in the expression of several snoRNAs have been reported.

### snoRNAs are abnormally expressed in cardiac hypertrophy

The pathological changes associated with cardiac hypertrophy may be closely related to snoRNAs, and abnormal expression of snoRNAs in hypertrophic hearts has been reported. snora48 is the most abundant snoRNA transcript in the rat heart and is predicted to direct pseudouridylation of 28S ribosomal RNA in the biogenesis of large ribosomal subunits. However, in the hypertrophic heart, snora48 is down-regulated. Therefore, dysregulation of snora48 may be harmful to cardiac ribosomal biogenesis.^18^ Wang et al found that in Angiotensin II (AngII)-infused mouse hearts, enhancer of zeste 2 (EZH2) could bind to multiple snoRNAs, including snora7a, snora31, snora33, snora47, snora68, snord15a, and snord104. Among them, snora33 has the highest interaction intensity with EZH2. The interaction between EZH2 and snoRNAs might contribute to the modulation of tRNA or rRNA maturation as well as ribosome assembly.^19^ Additionally, snoRNAs may also have a potential regulatory role in hypertrophic cardiomyopathy, a type of inherited heart disease. James et al conducted transcriptome analysis of extracellular vesicle (EV) cargoes extracted from human-induced pluripotent stem cell-derived cardiomyocytes (hiPSC-CMs) and found that the levels of 12 snoRNAs differed between EVs from wild-type and hypertrophic cardiomyopathy hiPSC-CMs, including ten C/D Box snoRNAs and two H/ACA Box snoRNAs. These snoRNAs are predicted to play roles in alternative splicing, post-translational modification, and the pathogenesis of hypertrophic cardiomyopathy through metabolic pathway regulation. Moreover, the levels of snoRNAs in EVs derived from hypertrophic cardiomyopathy hiPSC-CMs further changed when cardiac load increased, while such changes were not observed in EVs derived from wild-type hiPSC-CMs.^17^^,^^20^ In the *Drosophila melanogaster* model of feline hypertrophic cardiomyopathy established by Tallo et al, dysregulation of 23 snoRNAs was detected.^21^ These results suggest that snoRNAs are potential and important regulators of cardiac hypertrophy.

### snoRNAs are associated with IHD

Although there are limited investigations into the association between snoRNAs and IHD, some research has found that a correlation exists, and snoRNAs may hold value as dual diagnostic markers and treatment targets in IHD. One study demonstrated that plasma levels of SNORD113.2 in patients with ST-elevation MI were twice as high on day 4 post-hospitalization compared with day 30.^22^ The increased plasma concentrations of SNORD113.2 and SNORD114.1 correlate with platelet activation, an important prognostic factor for MI.^17^^,^^23^ In addition, Schena et al found that injection of cortical bone stem cells and cortical bone stem cell-derived exosomes into the ischemic region of infarcted hearts reduced infarct size, possibly mediated by a reduction in snoRNA levels. snoRNAs constitute a critical component in preserving ribosome stability. The decrease in snoRNAs induces ribosome instability and altered translation, leading to impaired gene translation in cardiac fibroblasts.^24^ Collectively, these results suggest that the relationship between snoRNAs and IHD deserves more attention.

### snoRNAs are potential regulators of HF

Changes in snoRNAs may play a significant role in HF. Håkansson et al found that single-nucleotide polymorphisms in snoRNA clusters were strongly linked to HF. Further studies found that snoRNAs in the 14q32 region were most highly expressed in end-stage failing human hearts. Moreover, 14q32 snoRNAs bind to Fibrillarin, a key enzyme involved in 2′-O-Me of target RNA. Given that miRNAs are subject to Fibrillarin-dependent 2′-O-Me and that 14q32 snoRNAs can bind to the miRNA processor protein argonaute RISC component 1 (AGO1), the authors speculate that 14q32 snoRNAs may target miRNA precursors to modulate their modification.^22^ Lin et al identified 4988 differentially methylated genes between the sham and HF groups in a rat model of ischemic HF, including snoRNAs, highlighting their potential role in this condition. DNA methylation occurs in many snoRNAs, but the underlying mechanisms in HF remain unclear.^25^ These findings indicate that snoRNAs play a biologically significant role in HF, warranting further studies to elucidate their molecular mechanisms.

From existing studies, we know that snoRNAs are differentially expressed in heart disease, which may be related to their function, but the specific mechanisms remain unclear. Based on current research, we hypothesize that snoRNAs may contribute to heart disease by mediating 2′-O-Me or pseudouridylation modifications of target RNAs, interacting with proteins, or performing other potential non-canonical functions, thereby regulating ribosome biogenesis or other biological processes. Unraveling the cardiopathological mechanisms of snoRNAs represents an important research frontier, which could provide new insights for diagnosis and treatment.

## piRNAs

### Characterization, biosynthesis, and function of piRNAs

Originally discovered in *D. melanogaster* germ cells, piRNAs (24–35 nt in length) derive their name from their interaction with the P-element induced wimpy testis (PIWI) protein.^5^^,^^9^^,^^26^ piRNAs have a 2′-O-methylated 3′end, while the 5′end usually shows a uridine bias.^9^ Although piRNA expression was originally thought to be confined to germ cells, contemporary studies have identified their presence in somatic cell populations, such as neural, cardiac, and hepatic tissues.^9^

Most piRNAs originate from defined genomic regions harboring piRNA clusters that contain transposable elements (TEs). These piRNA clusters can be categorized into single-stranded and double-stranded clusters.^27^ The single-strand piRNA clusters are the most widely distributed, with a transcription pathway similar to classical transcription, and the resulting transcripts can undergo normal splicing. However, double-stranded piRNA clusters are predominantly found in germ cells and lack defined promoters, and the newly generated precursor transcripts cannot undergo further post-transcriptional modifications. Instead, they are exported from the nuclear compartment into the cytoplasmic space for further processing into mature piRNAs.^27^

**Figure 2:**
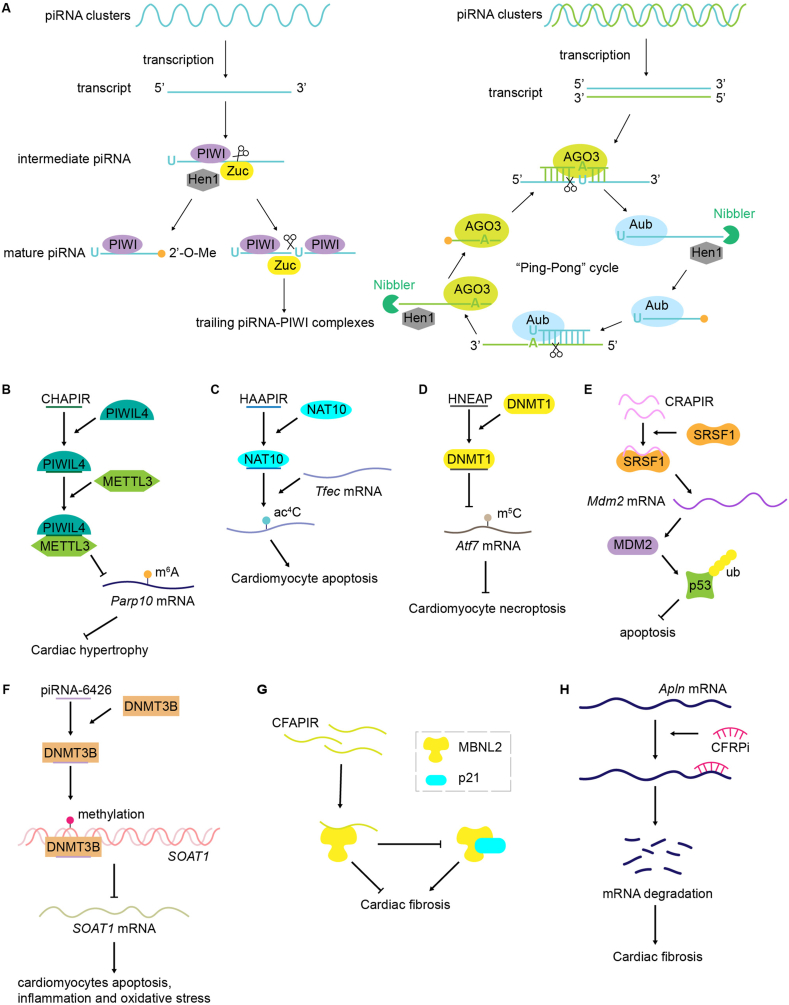
Biosynthesis pathway of piRNA and its mechanism in heart disease. **(A)** Biosynthetic pathway of piRNAs. Left: The primary pathway. Right: The secondary pathway, also known as the “Ping-Pong” cycle. In somatic cells, piRNAs are synthesized mainly through the primary pathway, while in germ cells, both the primary pathway and the secondary pathway are functional. **(B–H)** The mechanism of piRNAs in heart disease. The piRNAs currently identified in heart disease involve four main mechanisms: regulating epigenetic modifications through interactions with epigenetic modifiers (B–D, F), affecting the stability of downstream mRNAs and proteins by interacting with target proteins (E), competing for protein binding (G), and targeting the 3′ untranslated region (UTR) of mRNA to silence genes (H). Examples are shown below. (B) CHAPIR prevents METTL3 from binding to *Parp1*0 mRNA by capturing METTL3, reducing the level of N^6^-methyladenosine (m^6^A) modification on *Parp1*0 mRNA, and leading to cardiac hypertrophy. (C) HAAPIR binds to NAT10 and increases N^4^-acetylcytidine (ac^4^C) modification of the *Tfec* mRNA transcript, promoting its expression and ultimately leading to cardiomyocyte apoptosis. (D) HNEAP binds to DNMT1 and weakens the 5-methylcytosine (m^5^C) methylation of the *Atf7* mRNA transcript, promoting *Atf7* expression and thereby leading to cardiomyocyte necrosis. (E) CRAPIR regulates the stability of MDM2 mRNA and protein by binding to SRSF1, thereby promoting the ubiquitination of p53 and reducing apoptosis. (F) piRNA-6426 mediates *SOAT1* promoter methylation via DNMT3B recruitment, inhibiting *SOAT1* expression and preventing cardiomyocyte damage. (G) In cardiac fibroblasts, CFAPIR competes with p21 for binding to MBNL2. When CFAPIR binds to MBNL2, it inhibits cardiac fibrosis, whereas p21 binding to MBNL2 promotes cardiac fibrosis. (H) CFRPi silences *Apln* mRNA by targeting its 3′UTR, thereby promoting cardiac fibrosis. Figure created with Adobe Illustrator.

piRNA biosynthesis involves two stages: the primary pathway and the secondary pathway, with the secondary pathway known as the “Ping-Pong” cycle (Fig. 2A).^28^^,^^29^ Taking *D. melanogaster* as an example, piRNAs are mainly produced in somatic cells through the primary pathway, which involves first being transcribed from single-stranded piRNA clusters and then exported to Yb bodies after alternative splicing. In the Yb body, the piRNA precursor is cleaved by nuclease and subsequently binds to the PIWI protein, triggering translocation to the mitochondrial outer membrane, where it is further cleaved by Zucchini (Zuc). The resulting fragment is 2′-O-methylated by Hen methyltransferase 1 (Hen1) and then transported into the nucleus, while the remaining piRNA precursors can be reloaded onto PIWI proteins and cleaved again, resulting in a series of trailing piRNA–PIWI complexes (Fig. 2A Left).^28^^,^^29^ Germ cells utilize both the primary and secondary pathways. The primary pathway shares core features with its somatic counterpart, differing in that piRNA precursors originate from double-stranded piRNA clusters before being transported to nuage. The secondary pathway requires Aubergine (Aub) and Argonaute 3 (AGO3) to cleave TE mRNAs and piRNA cluster precursor transcripts in a complementary manner, and the resulting pre-piRNA is trimmed by Nibbler and 2′-O-methylated by Hen1 to become mature piRNA (Fig. 2A Right).^28^^,^^29^ piRNA biosynthesis is conserved across species, and a similar mechanism is used for piRNA synthesis in mice.^28^ The biogenesis of piRNA in humans has not been extensively studied, but research has revealed four human PIWI homologs: PIWIL1 (HIWI), PIWIL2 (HILI), PIWIL3 (HIWI3), and PIWIL4 (HIWI2).^9^^,^^27^

The most typical role of piRNAs is to facilitate PIWI-mediated transposon repression, thereby protecting genome integrity. Almost all animals use piRNAs to protect their genomes from transposon activity.^9^ However, the discovery of some piRNAs derived from non-transposable element sequences suggests that their functions may not be limited to silencing TEs.^27^ For example, they mediate gene silencing by targeting 3′ untranslated region (UTR).^30^ Additionally, piRNAs can regulate gene expression by recruiting epigenetic regulators.^27^

Although the roles of most piRNAs in somatic cells have not been well characterized, it has been found that an abundant population of piRNAs is detected in the heart and exhibits unique characteristics compared with those in germ cells. The piRNAs identified in the heart are approximately 24–25 nt in length, displaying a stronger 10A bias and a weaker 1U bias. Genomic mapping of cardiac piRNAs demonstrated predominant localization (> 90%) to a single 172 kb region on chromosome 14 [chr14:46693580-46865522 (+)].^31^ In recent years, the molecular mechanisms of piRNAs in heart disease have been gradually elucidated, including their roles in interactions with proteins and gene silencing through mRNA targeting, which has significantly enhanced our understanding of their functions.^30^^,^^32^, ^33^, ^34^ Next, we will highlight recent research progress concerning piRNA involvement in cardiac hypertrophy, IHD, and HF.

### Functions of piRNAs in cardiac hypertrophy

The expression profiles of piRNAs show significant and distinct differences between hypertrophic and normal cardiac tissue.^31^ The role of cardiac-hypertrophy-associated piRNA (CHAPIR), which is up-regulated in hypertrophic hearts of mice, has been extensively studied.^32^ Both *in vivo* and *in vitro* studies have demonstrated that CHAPIR is a critical regulator of cardiac hypertrophy. Overexpression of CHAPIR markedly aggravates cardiac hypertrophy, while its knockdown substantially suppresses the hypertrophic process.^32^ Further studies have revealed that CHAPIR interacts with methyltransferase 3 (METTL3). Although this interaction does not alter *Mettl3* expression, it inhibits METTL3 activity, leading to reduced N^6^-methyladenosine (m^6^A) methylation of poly (ADP-ribose) polymerase family, member 10 (*Parp10*) mRNA and subsequent up-regulation of its expression (Fig. 2B). PARP10, an adenosine diphosphate (ADP)-ribosyltransferase, catalyzes the mono-ADP-ribosylation of glycogen synthase kinase 3 beta (GSK3β), thereby suppressing its kinase activity. This suppression results in the accumulation of nuclear factor of activated T cells 4 (NFATC4) in the nucleus, driving the progression of pathological cardiac hypertrophy.^32^ Additionally, diabetic cardiomyopathy can induce significant pathological cardiac hypertrophy, which can be mitigated by overexpression of piR112710.^35^ Collectively, these findings demonstrate that piRNAs play a crucial regulatory role in cardiac hypertrophy and may serve as promising therapeutic targets.

### Regulation of IHD by piRNAs

Currently, several piRNAs associated with IHD and their mechanisms of action have been discovered. piRNAs display distinct expression patterns in serum samples obtained from acute myocardial infarction cases, with most piRNAs showing up-regulated expression.^36^ Mechanistically, piRNAs influence cardiomyocyte death through epigenetic regulation. For example, heart-apoptosis-associated piRNA (HAAPIR) increases N^4^-acetylcytidine (ac^4^C) modification and expression of transcription factor EC (*Tfec*) mRNA by binding to N-acetyltransferase 10 (NAT10) and regulating its activity (Fig. 2C). Subsequently, TFEC acts as a transcription factor to bind to the promoter region of BCL2-interacting killer (*Bik*), a pro-apoptotic protein gene, promoting its expression and leading to apoptosis. Therefore, the absence of HAAPIR can inhibit cardiomyocyte apoptosis and reduce infarct size.^33^ Similarly, heart necroptosis-associated piRNA (HNEAP) binds to DNA methyltransferase 1 (DNMT1) and reduces its activity, thereby weakening the 5-methylcytosine (m^5^C) methylation of activating transcription factor 7 (*Atf7*) mRNA and increasing its expression (Fig. 2D). ATF7 then binds to the promoter of charged multivesicular body protein 2A (*Chmp2a*), a necrosis inhibitor, to suppress its expression, resulting in the progression of cardiomyocyte necrosis. Thus, HNEAP knockout inhibits cardiomyocyte necrosis, reduces infarct size, and improves cardiac function.^34^ piRNAs also stabilize downstream mRNAs and proteins by interacting with target proteins. A notable example is cardiac regeneration-associated PIWI-interacting RNA (CRAPIR), which interacts with serine and arginine-rich splicing factor 1 (SRSF1) to stabilize murine double minute 2 (*Mdm2*) mRNA and inhibit MDM2 protein degradation. The consequent increase in MDM2 promotes the ubiquitination and degradation of p53, ultimately providing a protective effect against myocardial I/R injury (Fig. 2E).^37^ In summary, the expression patterns and regulatory mechanisms of piRNAs in IHD have been identified, and further efforts are warranted to develop them as diagnostic markers or therapeutic targets for the disease.

### Mechanisms of piRNAs in HF

piRNAs are also differentially expressed in serum EVs of patients with HF. Contrary to the expression pattern of piRNAs in the serum of patients with acute myocardial infarction, most piRNAs in patients with HF are down-regulated.^38^ Some mechanisms by which piRNAs regulate HF progression have been revealed. For example, piRNA-6426, which is down-regulated in HF patients with ischemic etiology, interacts with DNA methyltransferase 3 beta (DNMT3B) and positively regulates its activity. The promoter region of sterol o-acyltransferase 1 (*SOAT1*) is a target of DNMT3B (Fig. 2F). The down-regulation of piRNA-6426 reduces DNMT3B enrichment and the methylation level in the *SOAT1* promoter region. This results in increased *SOAT1* expression, exacerbating oxidative stress, inflammation, and cardiomyocyte apoptosis (Fig. 2F).^39^ Overexpression of piRNA-6426 has been shown to alleviate myocardial dysfunction in rats, highlighting its candidacy for a novel target for HF diagnosis and treatment.^39^ Furthermore, anti-fibrotic strategies in cardiac remodeling represent key approaches to limit HF advancement, with piRNAs contributing to these pathological events. For instance, cardiac fibrosis-associated piRNA (CFAPIR) inhibits the activation of the transforming growth factor beta 1/SMAD family member 3 (TGF-β1/SMAD3) signaling pathway and fibrosis progression by competing with p21 for binding to muscleblind-like splicing regulator 2 (MBNL2) (Fig. 2G).^40^ Another piRNA, cardiac fibrosis-related piRNA (CFRPi), showed marked elevation in HF specimens from mice, humans, and pigs, with its expression gradually increasing parallel to the progression of pressure overload-induced HF. Mechanistically, CFRPi binds to the 3′UTR region of apelin (*Apln*) mRNA, causing gene silencing and further promoting the activation of the phosphoinositide 3-kinase/protein kinase B/mechanistic target of rapamycin (PI3K/AKT/mTOR) pathway, which subsequently drives cardiac fibrosis (Fig. 2H).^30^ Overall, piRNAs not only play an important regulatory role in the progression of HF but also hold potential as therapeutic targets and diagnostic markers in the future.

In general, the mechanisms by which piRNAs regulate heart disease include interacting with proteins and regulating their expression or activity, directly targeting mRNA to silence genes, and competing with proteins for binding to regulate signaling pathways (Fig. 2B–H). Although the mechanisms of piRNA involvement in heart disease are gradually being revealed, more research and clinical data are needed to develop drugs targeting piRNAs and piRNA-based therapies.

## tsRNAs

### Classification and function of tsRNAs

As we all know, tRNAs are important molecules that decode mRNA and participate in protein biosynthesis. However, mature tRNAs or their precursors can be cleaved into small fragments called tsRNAs through the action of various ribonucleases, such as angiogenin (ANG), ELAC2, RNase T2, RNase P, and RNASE1, with DICER also contributing to the processing of some tsRNAs.^10^^,^^41^, ^42^, ^43^, ^44^

**Figure 3:**
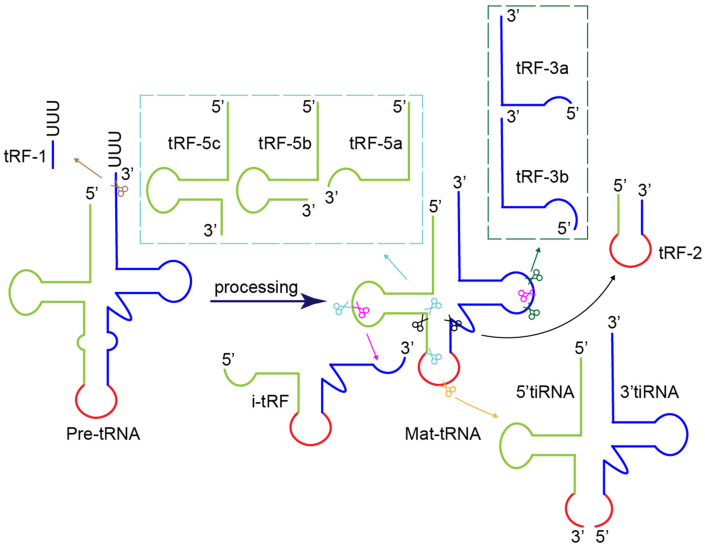
Classification of tsRNAs. tsRNAs, which include tiRNAs and tRFs, are processed from precursor or mature tRNAs. Specifically, tiRNAs are formed by cleavage at the anti-codon loop, yielding both 5′tiRNAs and 3′tiRNAs. Based on the cleavage site and length, tRFs can be further classified into tRF-5, tRF-3, tRF-1, tRF-2, and i-tRF. The different colors of scissors in the figure indicate different cleavage sites. Figure created with Adobe Illustrator.

Structurally, tsRNAs exist as two predominant forms: tRNA halves (tiRNAs) and tRNA-derived fragments (tRFs).^5^^,^^10^^,^^45^ Among these, tiRNAs can be further classified into 5′tiRNAs and 3′tiRNAs. These fragments are typically 29–50 nt in length and are usually produced by the cleavage of the anti-codon loop by ANG (Fig. 3). By comparison, tRFs are smaller fragments, approximately 14–30 nt in length, generated from mature or precursor tRNAs through Dicer-dependent processes. Based on their origin within the tRNA, tRFs can be further subdivided into tRF-5, tRF-3, tRF-1, tRF-2, and i-tRF.^5^^,^^10^^,^^45^ Additionally, according to the length of the fragment, tRF-5 can be categorized into tRF-5a, tRF-5b, and tRF-5c; tRF-3 into tRF-3a and tRF-3b (Fig. 3).^45^

The functions of tsRNAs are extensive and complex, and they are known to be involved in many biological processes, such as protein translation regulation, mRNA silencing, epigenetic modification, and apoptosis inhibition.^5^^,^^10^^,^^45^ Furthermore, the high expression of tsRNAs in plasma EVs suggests that they may play a role in mediating cell communication.^41^^,^^45^

tsRNAs are expressed in the heart. Moreover, the expression of tsRNAs can be induced by pathological stress, which is closely related to heart disease, suggesting that they may serve as potential regulators of heart disease.^41^^,^^46^

### tsRNAs are regulators of cardiac hypertrophy

Emerging research implicates tsRNAs in cardiac hypertrophy pathogenesis, primarily through 3′UTR targeting and subsequent gene silencing. In the rat model of isoproterenol-induced cardiac hypertrophy, tRFs were found to be highly enriched in the hypertrophic heart. Overexpression of tRFs1 and tRFs2 in H9c2 cells up-regulated the hypertrophy markers and increased the surface area of cardiac cells. Further studies found that tRFs1 inhibited TIMP metallopeptidase inhibitor 3 (*Timp3*) expression by directly targeting the 3′UTR. Knocking out *Timp3* has been shown to cause cardiac fibrosis and hypertrophy. Therefore, tRFs1 promotes cardiac hypertrophy by down-regulating *Timp3*.^47^ Interestingly, tRFs could serve as a newly identified molecular basis for the intergenerational inheritance of cardiac hypertrophy. Some tRFs enriched in the heart are also abundant in sperm, and F_1_ offspring of cardiac hypertrophy rats show elevated expression of hypertrophy markers and symptoms of hypertrophic heart compared with controls. However, considering the side effects of isoproterenol on spermatogenesis, further research is needed to validate this process.^47^ tRF-16-R29P4PE was significantly down-regulated in plasma samples from patients with pathological cardiac hypertrophy. Mechanistic studies demonstrated its functional role through targeting the 3′UTR of paired basic amino acid cleaving system 4 (*PACE4*) mRNA, with *PACE4* silencing effectively suppressing AngII-induced cardiomyocyte hypertrophy.^48^ Beyond these findings, multiple tsRNAs, including the down-regulated tRF-21-NB8PLML3E, show altered expression patterns in the plasma of cardiac hypertrophy patients. Nevertheless, the mechanistic underpinnings of these alterations remain unclear.^49^ Additional studies will be essential to expand our current understanding of tsRNA-mediated regulatory mechanisms underlying cardiac hypertrophy development.

### tsRNAs play an important role in IHD

Current research indicates that tsRNAs are closely associated with IHD, either by interacting with proteins or by mediating gene silencing through base pairing. Wang et al identified a 5′tiRNA, AS-tDR-001449, which promotes cardiomyocyte apoptosis and was significantly up-regulated in the hearts of mice with MI. This 5′tiRNA is generated through hypoxia-inducible factor 1-alpha (HIF1A)/ANG axis-mediated cleavage of the anti-codon loop in tRNA-Val-CAC under hypoxia-induced conditions. Mechanistically, AS-tDR-001449 binds to human antigen R (HuR) and enhances its interaction with *p5*3 mRNA, thereby stabilizing *p53* transcripts and promoting cell apoptosis.^50^ Hao et al found that tsRNAs expression patterns were altered in rat models of MI, and the levels of both NAT10 and ac^4^C acetylation were increased in fibrotic tissues after infarction. The acetyltransferase NAT10 plays a central role in mediating acetylation modifications, and ac^4^C is a conserved modification of multiple RNA types, which is closely related to the promotion of protein translation and mRNA stability. Therefore, it is likely that NAT10 regulates the fibrotic process through ac^4^C acetylation modification of target RNA. Consistent with this view, the authors found that the mRNA expression and acetylation levels of early growth response 3 (*Egr3*), an important molecule that promotes myocardial fibrosis, were significantly increased.^51^ Among the altered tsRNAs, the expression of tsr007330, a 5′tiRNA, is down-regulated. Notably, tsr007330 overexpression results in decreased expression of *Nat10* and *Egr3*, along with diminished ac^4^C modification. More importantly, bioinformatic analysis revealed potential base-pairing interactions between tsr007330 and *Nat1*0 mRNA. Therefore, tsr007330 may regulate post–MI fibrosis through NAT10-mediated ac^4^C acetylation modification of *Egr3* mRNA, suggesting its potential as an effective therapeutic target.^51^ These studies have deepened our understanding of how tsRNAs regulate IHD. Although the research remains preliminary, it highlights the indispensable regulatory role of tsRNAs in IHD pathogenesis.

### tsRNAs may contribute to HF progression

While studies of tsRNAs in HF are still in early stages, their pathological relevance is gradually recognized. Compared with the control group, tsRNAs are differentially expressed in the epicardial adipose tissue of acute heart failure patients. Two down-regulated tRFs (tRF-Tyr-GTA-010 and tRF-Tyr-GTA-011) target genes primarily involved in Ca^2+^ transport, nitric-oxide synthase binding, and cellular components of the somatodendritic compartment. Furthermore, the signaling pathways associated with these tRFs are predominantly enriched in sphingolipid signaling, adrenergic signaling in cardiomyocytes, and the mRNA monitoring pathway. Collectively, these findings demonstrate that abnormal tRF regulation and consequent pathway disturbances may drive HF pathogenesis.^52^

Although current studies have shown that tsRNAs are closely associated with heart disease, our understanding of their regulatory mechanisms remains relatively preliminary. Despite this, based on current evidence, it is understood that tsRNAs may perform their functions by targeting RNA or interacting with proteins. The clearly defined mechanisms are listed in Table 1. Future investigations should aim to unravel the molecular basis of tsRNAs in this context, including how they are produced and how they function.

**Table 1:** Mechanisms of tsRNAs in heart diseases.

tsRNA	Target RNA or interacting protein	Mechanism and function	Expression of tsRNA in heart disease	Reference
tRFs1	*Timp3*	Inhibiting *Timp3* expression and promoting cardiac hypertrophy	Up-regulated in cardiac hypertrophy	^47^
tRF-16-R29P4PE	*PACE4*	Inhibiting *PACE4* expression and alleviating cardiac hypertrophy	Down-regulated in cardiac hypertrophy	^48^
AS-tDR-001449	HuR	Enhancing the interaction between HuR and *p5*3 mRNA, increasing the stability of *p5*3 mRNA, and promoting apoptosis	Up-regulated in myocardial infarction	^50^
tsr007330	*Nat10*	Down-regulating *Nat10* expression, reducing *Egr3* mRNA ac^4^C modification, and alleviating myocardial fibrosis	Down-regulated in myocardial infarction	^51^

## circRNAs

### Characterization, biosynthesis, function, and degradation of circRNAs

As the name suggests, circRNAs form covalently linked circular transcripts lacking terminal modifications (5′ cap structure or 3′ polyadenylated tail), which makes them more stable.^5^^,^^12^^,^^53^ Based on their sequence composition, circRNAs exist in three structural variants: exonic circRNAs (EcircRNAs), circular intronic RNAs (ciRNAs), and exon-intron circRNAs (EIciRNAs).^54^

**Figure 4:**
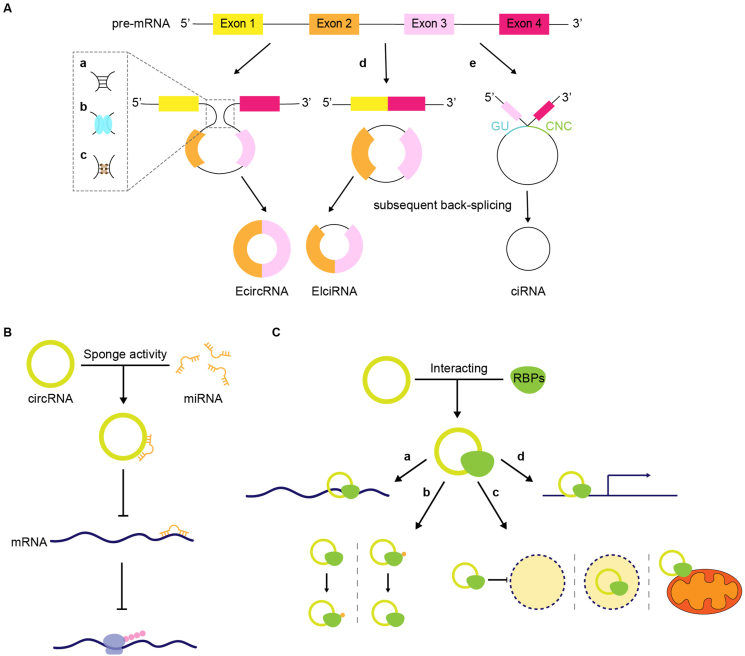
Biosynthesis of circRNA and its mechanism in heart disease. **(A)** Biosynthesis of circRNA. Several back-splicing mechanisms are involved in circRNA formation: **(a)** intron pairing facilitated by reverse complementary sequences; **(b)** intron pairing mediated by RNA-binding proteins (RBPs); **(c)** intron pairing mediated by m^6^A modification; **(d)** exon skipping; **(e)** lariat formation. **(B, C)** Two primary mechanisms of circRNAs in heart disease. (B) The circRNA–miRNA–mRNA network. MiRNAs cause gene silencing through their interactions with mRNAs. CircRNAs act as molecular sponges for miRNAs, blocking the binding of miRNAs to target mRNAs and thereby regulating gene expression. (C) The interaction between circRNAs and RBPs. This interaction can lead to multiple downstream events, such as: **(a)** mRNA stabilization; **(b)** modification of post-translational modifications on proteins (*e.g.*, writing or erasing modifications); **(c)** changes in protein subcellular localization (left: blocking nuclear entry; middle: promoting nuclear entry; right: inducing mitochondrial translocation); and **(d)** regulation of gene expression. Figure created with Adobe Illustrator.

The formation of circRNAs is based on the back-splicing of exon and/or intron rearrangements in pre-mRNA.^55^ Several mechanisms have been identified, including intron pairing facilitated by reverse complementary sequences, the participation of RNA-binding proteins (RBPs), m^6^A modification, exon skipping, and lariat formation (Fig. 4A).^54^^,^^55^ Among these, the first four mechanisms are commonly involved in the synthesis of circRNAs containing exon sequences. Through these mechanisms, the splicing sites gradually come closer, ultimately promoting the formation of circRNAs (Fig. 4A). However, in processes involving RBPs, these proteins may also inhibit circRNA biosynthesis.^53^, ^54^, ^55^ Lariat formation plays a crucial role in the synthesis of ciRNAs.^12^^,^^54^^,^^56^ This process involves two consensus motifs: a GU-rich element (GU) near the 5′ segment and a C-rich element (CNC) near the branchpoint site. These two conserved motifs are essential for the lariat structure to escape debranching enzyme-mediated degradation (Fig. 4A).^54^^,^^57^

circRNAs have a variety of functions in cells. Among them, the most well-known is their ability to target miRNAs through the “sponge effect”, thereby promoting downstream protein expression. A single circRNA may target multiple miRNAs.^5^^,^^12^^,^^54^ Additionally, circRNAs can interfere with post-transcriptional regulation and mRNA translation by competing with mRNAs for binding to RBPs. They are also capable of interacting with various proteins, creating specific circRNA-protein complexes that alter the functional properties of the bound proteins. Furthermore, through interaction with RNA polymerase II, circRNAs can promote the transcriptional activation of their host genes.^5^^,^^12^ Moreover, circRNAs are exported to the extracellular space, where they may participate in cellular communication.^12^^,^^56^ Notably, although most circRNAs cannot be translated, a few can encode microproteins through internal ribosome entry site (IRES)-dependent translation or m^6^A-dependent translation. These microproteins typically lack functional domains compared with typical proteins and are thought to play roles in regulating alternative protein complexes.^5^^,^^12^^,^^54^

Although circRNAs are more stable than mRNAs, they can be degraded through a variety of mechanisms. These mechanisms include miRNA-mediated degradation, m^6^A modification coupled with decoding protein recognition, and endonuclease-mediated degradation. In addition, the UPF1/G3BP1 pathway may be involved in circRNA degradation, and its absence leads to an increased level of circRNAs. Silencing of GW182 and its human counterparts also increases circRNA levels, suggesting their potential involvement in the degradation process.^54^

In heart disease, multiple mechanisms involving circRNAs have been revealed. Among them, the most studied is the role of circRNAs through the circRNA–miRNA–mRNA network. In addition, the interaction between circRNAs and RBPs, as well as the regulatory mechanisms of circRNA production, have also been gradually elucidated. Below, we describe in detail the mechanisms involving circRNAs in cardiac hypertrophy, IHD, and HF.

### circRNAs are important regulators of cardiac hypertrophy

circRNA expression is either up-regulated or down-regulated in cardiac hypertrophy, which involves multiple mechanisms. On the one hand, the generation of circRNAs can be regulated. The promoter region of myosin IXa (*Myo9a*), the parental gene of circRNA_000203, contains nuclear factor kappa B (NF-κB) recognition elements. Thus, NF-κB signaling mediates AngII-induced up-regulation of circRNA_000203.^58^ DExH-box helicase 9 (DHX9), a DNA duplex helicase that inhibits circRNA production, may be involved in circSMAD3 production, as AngII promotes DHX9 protein levels and decreases circSMAD3 expression, while silencing *DHX9* promotes circSMAD3 expression.^59^ As a histone methyltransferase, nuclear receptor binding SET domain protein 2 (NSD2) catalyzes histone H3 lysine 36 (H3K36) methylation, generating mono-, di-, and/or tri-methylated states (H3K36me1/2/3). Among these modifications, NSD2-mediated H3K36me2 activates the expression of cms small ribosomal subunit 1 (*Cmss1*), leading to the elevated expression of circCmss1 and promoting ventricular remodeling.^60^ On the other hand, the stability and degradation of circRNAs are also regulated. Under the stimulation of isoproterenol, the m^6^A eraser alkylated DNA repair protein alkB homolog 5 (ALKBH5) is down-regulated. As a result, an elevated m^6^A modification level is detected in circPan3, which ultimately leads to its destabilization.^61^ Moreover, in addition to regulating circRNA expression and degradation within cells themselves, these differentially expressed circRNAs may exist in EVs secreted by other cells and function through cell-to-cell communication.^62^

At present, the identified mechanisms involving circRNAs in cardiac hypertrophy mainly fall into two categories: circRNA–miRNA–mRNA network and the interaction between circRNAs and RBPs. We have listed the circRNA–miRNA–mRNA network and the circRNA-RBP interactions in cardiac hypertrophy in Table 2 and Table 3, respectively. The regulatory targets of these two mechanisms primarily include the host genes of circRNAs or other hypertrophy-associated proteins.^63^, ^64^, ^65^, ^66^ These regulated targets further influence downstream events, such as transcriptional activation or inhibition, regulation of signaling pathways, and actin polymerization, thereby promoting or inhibiting cardiac hypertrophy.^58^^,^^59^^,^^64^^,^^67^^,^^68^ Notably, both mechanisms may work together. For example, circ-SIRT1 regulates sirtuin 1 (*SIRT1*) expression by targeting miR-3681-3p and miR-5195-3p. Simultaneously, circ-SIRT1 induces SIRT1 deubiquitination by recruiting ubiquitin-specific peptidase 22 (USP22), thereby stabilizing the SIRT1 protein.^69^ Similarly, circ_0001052 acts as a competitive endogenous RNA (ceRNA) for homeodomain-interacting protein kinase 3 (*Hipk3*) by targeting miR-148a-3p and miR-124-3p. In addition, circ_0001052 stabilizes *Hipk3* mRNA by recruiting SRSF1. Thus, circ_0001052 regulates *Hipk3* expression through both mechanisms.^63^

**Table 2:** Identified circRNA–miRNA–mRNA networks in cardiac hypertrophy.

circRNA	miRNA	mRNA	Function	Expression of circRNA in cardiac hypertrophy	Reference
circHIPK3	miR-185-3p	*Casr*	Promoting cardiac hypertrophy	Up	^65^
circRNA_0068481	miR-646 miR-570 miR-885	*EYA3*	Promoting right ventricular hypertrophy in patients with pulmonary arterial hypertension (PAH)	Up	^70^
circRNA_000203	miR-26b-5p miR-140-3p	*Gata4*	Promoting cardiac hypertrophy	Up	^58^
circNfix	miR-145-5p	*Atf3*	Inhibiting cardiac hypertrophy	Down	^71^
circ-SIRT1	miR-3681-3p miR-5195-3p	*SIRT1*	Inducing myocardial autophagy and inhibiting cardiac hypertrophy	Down	^69^
circ_0001859	miR-29b-3p	*Ctnnb1*	Activating the LEF1/IGF-2R pathway and promoting ambient fine particulate matter (PM_2.5_) exposure-induced cardiac hypertrophy	Up	^67^
circ_0001006	miR-214-3p	*Pak6*	Promoting cardiac hypertrophy	Up	^72^
circ_0001052	miR-148a-3p miR-124-3p	*Hipk3*	Promoting cardiac hypertrophy	Up	^63^
circCacna1c	miR-29b-2-5p	*Nfatc1*	Promoting the expression of hypertrophic genes	Up	^64^
circPan3	miR-320-3p	*Hsp20*	Alleviating cardiac hypertrophy	Down	^61^
circRNA CHRC	miR-431-5p	*Klf15*	Alleviating cardiac hypertrophy	Down	^73^
circ_Larp4b	miR-298-5p	*Mef2c*	Promoting cardiac hypertrophy	Up	^74^
circ_0018553	miR-4731	*SIRT2*	Circ_0018553 is enriched in extracellular vesicles (EVs) derived from endothelial progenitor cells (EPCs) and alleviates cardiac hypertrophy	Down	^62^

**Table 3:** circRNAs interacting proteins in cardiac hypertrophy.

circRNA	Interacting protein	Mechanism	Expression of circRNA in cardiac hypertrophy	Reference
circ-SIRT1	USP22	Inducing SIRT1 deubiquitination and stabilizing SIRT1 protein	Down	^69^
circ-TLR4	FUS	Stabilizing *TLR4* mRNA	Up	^66^
circ_0001052	SRSF1	Stabilizing *Hipk3* mRNA	Up	^63^
circ_005077	CYPA	Inhibiting CYPA degradation through the ubiquitin-proteasome system (UPS), promoting the interaction between CYPA and p47phox, enhancing NADPH oxidase activity, and subsequently inducing ferroptosis	Up	^75^
circCmss1	EIF4A3	Inducing the expression of *TfR1* and promoting ferroptosis	Up	^60^
CHACR	CPT1b	Reducing the degradation of the CPT1b protein, thereby lowering l-carnitine levels and inhibiting the Jak2/Stat3 signaling pathway	Down	^76^
circ-0001283	MYL3	Preventing the ubiquitination of MYL3 and enhancing autophagy	Up	^77^
circSMAD3	YBX1	Stabilizing YBX1 and promoting its binding to SMAD3 in the nucleus, disrupting the TGF-β1/SMAD3 signaling pathway, and alleviating cardiac hypertrophy and fibrosis	Down	^59^
circYap	TPM4 ACTG	Increasing the interaction between TPM4 and ACTG, thereby inhibiting actin polymerization and cardiac fibrosis	Down	^78^
circITGa9	TPM3	Inducing actin polymerization and promoting cardiac fibrosis	Up	^68^

Of note, recent studies have revealed that microproteins encoded by circRNAs can promote cardiac hypertrophy progression. Specifically, circCDYL encodes a 100-amino acid microprotein (tCDYL-100) in an m^6^A-dependent manner. By competitively displacing chromodomain Y-like (CDYL) from RE1-silencing transcription factor (REST), tCDYL-100 disrupts the REST–CDYL–EHMT2 (euchromatic histone-lysine N-methyltransferase 2) complex, inducing cardiac hypertrophy.^79^ Given that some circRNAs can encode microproteins, future research on the role of these microproteins in heart diseases will enhance our understanding of circRNA-mediated mechanisms.

### Multiple circRNAs are closely associated with the progression of IHD

To date, multiple circRNAs display expression changes in IHD and have demonstrated potential as disease biomarkers.^80^, ^81^, ^82^ As research progresses, the regulatory mechanisms by which circRNAs are involved in IHD have been gradually uncovered.

The first mechanism is the regulation of circRNA generation and degradation. Several transcription factors have been found to be involved in this process, including GATA binding protein 4 (GATA4) and nuclear factor erythroid 2-related factor 2 (NRF2).^83^^,^^84^ In addition, the generation of circRNAs is regulated by feedback. circ-SNRK plays a cardioprotective role by regulating SNF-related kinase (SNRK) through sponging miR-33.^85^ NOVA alternative splicing regulator 1 (NOVA1) can interact with the flanking introns of pre-circ-SNRK to promote the formation of circ-SNRK, which is regulated by feedback from SNRK. Activated caspase 3 can degrade SNRK into two peptides, of which the 55 kDa cleavage peptide can enter the nucleus to interact with NOVA1. This interaction inhibits NOVA1 binding to introns, thereby blocking the cyclization of circ-SNRK. Exogenous overexpression of circ-SNRK can disrupt the feedback loop and enhance cardiac performance after MI in rats, highlighting its therapeutic candidacy.^85^ The m^6^A modification of circRNAs is critical for their stability. During myocardial I/R injury, the up-regulation of ALKBH5 eliminates the m^6^A modification on circDhx32. Consequently, circDhx32 cannot be effectively recognized and bound by YTHDF2 and YTHDF1, which promotes its stability and nuclear retention, respectively.^86^

In terms of the downstream mechanisms of circRNAs, multiple circRNA–miRNA–mRNA interactions associated with IHD were identified and listed in Table 4. These differentially expressed circRNAs originate either from resident cardiac cells (including cardiomyocytes, cardiac fibroblasts, and vascular endothelial cells) or from EVs secreted by non-cardiac cell types. For example, circUbe3a is derived from small extracellular vesicles of M2 macrophages, while circRNA_0002113 is present in EVs derived from mesenchymal stem cells.^87^^,^^88^ These circRNAs act as molecular sponges for miRNAs, thereby regulating the expression of their target genes. Notably, the same circRNA may play different regulatory roles by targeting different genes. For instance, circPAN3 regulates apoptosis through the miR-221/phosphatase and tensin homolog (PTEN)/PI3K/AKT pathway and autophagy through the miR-221/forkhead box O3 (Foxo3)/autophagy-related 7 (ATG7) pathway.^89^^,^^90^ In addition to functioning as miRNA sponges, circRNAs may also regulate miRNA expression through other mechanisms, such as methylation and promoting miRNA maturation.^91^^,^^92^ In AC16 cells, overexpression of the mitochondrial fission and apoptosis-related circRNA (MFACR) led to reduced miR-125b expression accompanied by increased methylation of the miR-125b gene locus, suggesting an epigenetic silencing mechanism mediated by MFACR.^91^ In MI patients, both circRNA ACAP2 and miR-532 are increased in plasma and exhibit a positive correlation. Overexpression of ACAP2 in cardiomyocytes increased the level of mature miR-532 but did not affect the expression of miR-532 precursors. Moreover, treatment with miR-532 inhibitors reduced the effect of ACAP2 overexpression. This suggests that ACAP2 may be involved in miR-532 maturation, although the specific mechanism remains unclear.^92^

**Table 4:** Identified circRNA-miRNA-mRNA networks in ischemic heart disease.

circRNA	miRNA	mRNA	Function	Expression of circRNA in ischemic heart disease	Reference
circ-Ttc3	miR-15b-5p	*Arl2*	Protecting cardiomyocytes from MI-induced apoptosis	Up	^93^
circ_0068655	miR-498	*PAWR*	Promoting cardiomyocyte apoptosis	Up	^94^
circMACF1	miR-500b-5p	*Emp1*	Inhibiting cardiomyocyte apoptosis and reducing myocardial injury	Down	^95^
circRNA 010567	miR-141	*Dapk1*	Impairing cardiomyocyte viability	Up	^96^
circPostn	miR-96-5p	*BNIP3*	Promoting cardiomyocyte damage induced by hypoxia/reoxygenation (H/R) treatment	Up	^97^
circRbms1	miR-92a	*Bcl2l11*	Promoting cardiomyocyte apoptosis and oxidative stress in ischemia-reperfusion (I/R) injury	Up	^98^
circRbms1	miR-742-3p	*Foxo1*	Aggravating hypoxia-induced cardiomyocyte injury	Up	^99^
circROBO2	miR-1184	*Tradd*	Promoting cardiomyocyte apoptosis	Up	^100^
circRNA_0002113	miR-188-3p	*Runx1*	circRNA_0002113 is present in EVs derived from mesenchymal stem cells; it promotes the nuclear translocation of RUNX1 and mediates apoptosis in anoxia-reoxygenation (A/R)-treated H9c2 cells by regulating the USP7/p53 pathway	Up	^88^
circ_0001206	miR-665	*Crkl*	Promoting cardiomyocyte activity and inhibiting apoptosis	Down	^101^
circTRRAP	miR-370-3p	*PAWR*	Promoting apoptosis, inflammation, and oxidative stress	Up	^102^
circ_0007059	miR-378 miR-383	Unknown	Promoting cardiomyocyte apoptosis and inflammation	Up	^103^
circITGB1	miR-342-3p	*NFAM1*	CircITGB1 is derived from EVs; it regulates dendritic cell maturation and cardiac inflammation	Up	^104^
circ-USP39	miR-499b-5p	*ACSL1*	Promoting hypoxia-induced apoptosis and injury in cardiomyocytes	Up	^105^
circ_0008842	miR-574-5p	*CALML4*	Inhibiting H/R-induced apoptosis and increasing cell viability	Down	^106^
circDiaph3	miR-338-3p	*Srsf1*	Aggravating H/R-induced cardiomyocyte apoptosis and inflammation	Up	^107^
circ_0020887	miR-370-3p	*CYP1B1*	Promoting hypoxia-induced cardiomyocyte injury	Up	^108^
circ-Stt3b	miR-15a-5p	*Gpx4*	Circ-Stt3b is present in EVs derived from hypoxic-pretreated adipose-derived mesenchymal stem cells (ADSCs); it reduces ferroptosis and exerts a protective effect on the heart	Down	^109^
circ ACAP2	miR-29	Unknown	Promoting apoptosis	Up	^110^
circHelz	miR-133a-3p	*Nlrp3*	Promoting myocardial damage	Up	^111^
circJARID2	miR-9-5p	*Bnip3*	Promoting cardiomyocyte damage	Up	^112^
circPAN3	miR-221	*Pten*	Regulating apoptosis	Up	^89^
circANKIB1	miR-452-5p	*Slc7a11*	Alleviating hypoxia-induced cardiomyocyte injury	Down	^113^
circRNA Pum1_0014	miR-146a-5p	*Nf2*	Promoting cardiomyocyte apoptosis	Up	^114^
circDGKZ	miR-345-5p	*TLR4*	Promoting cardiomyocyte pyroptosis	Up	^115^
circ_ZNF512	miR-181d-5p	*Egr1*	Inhibiting autophagy and promoting myocardial I/R injury	Up	^116^
circ-NNT	miR-33a-5p	*Usp46*	Promoting pyroptosis and aggravating myocardial I/R injury	Up	^117^
circCHSY1	miR-24-3p	*HO1*	Protecting the heart against I/R injury	Up	^118^
circMIRIAF	miR-544	*WDR12*	Aggravating myocardial I/R injury	Up	^119^
circHIPK3	miR-20b-5p	*Atg7*	Aggravating autophagy and apoptosis during myocardial I/R injury	Up	^120^
circSNRK	miR-103-3p	*Snrk*	Promoting cardiac survival and functional recovery post–MI through regulation of GSK3β phosphorylation	Down	^121^
circFASTKD1	miR-106a	*LATS1* *LATS2*	Inhibiting the YAP pathway and angiogenesisIncreasing infarct size	Unknown	^122^
circERBB2IP	miR-145a-5p	*Smad5*	Promoting post–MI angiogenesis	Unknown	^123^
circCEBPZOS	miR-1178-3p	*PDPK1*	CircCEBPZOS is derived from EVs; it promotes angiogenesis and improves heart function	Down	^124^
circHipk3	miR-133a	*CTGF*	Activating endothelial cells and promoting angiogenesis	Unknown	^83^
circMACF1	miR-16-5p	*SMAD7*	Inhibiting the activation of cardiac fibroblasts and alleviating cardiac fibrosis	Down	^125^
circUbe3a	miR-138-5p	*Rhoc*	circUbe3a, present in small extracellular vesicles derived from M2 macrophages, promotes functional changes in cardiac fibroblasts and exacerbates fibrosis	Up	^87^
circPAN3	miR-221	*Foxo3*	Activating autophagy and promoting fibrosis through the upregulation of *Atg7* transcription	Up	^90^
circCDYL	miR-4793-5p	*App*	Promoting cardiomyocyte proliferation	Down	^126^

The interaction between circRNAs and their protein partners is one of the important mechanisms underlying their biological functions. The proteins that interact with circRNAs in IHD are listed in Table 5. These interacting proteins have various functions, including acting as suppressors of transcription factors, interfering with nuclear translocation, stabilizing mRNA, and influencing protein expression levels.^127^, ^128^, ^129^ The functions of these interacting proteins further influence downstream signaling, leading to either the progression of IHD or improved cardiac function.^127^^,^^129^

**Table 5:** circRNAs interacting proteins in ischemic heart disease.

circRNA	Interacting protein	Mechanism	Expression of circRNA in ischemic heart disease	Reference
CNEACR	HDAC7	Intervening in the nuclear entry of HDAC7 and promoting *Foxa2* transcription. Subsequently, FOXA2 inhibits *Ripk3* gene expression, thereby alleviating cardiomyocyte death.	Down	^130^
FEACR	NAMPT	Regulating cardiomyocyte ferroptosis through the NAMPT-Sirt1-FOXO1-*Fth1* signaling axis and protecting cardiac function from I/R injury	Down	^131^
hsa_circ_0000848	ELAVL1	Stabilizing *Smad7* mRNA and reducing cardiomyocyte apoptosis	Down	^128^
CDR1as	NAMPT	Inhibiting NAMPT expression at the protein level, leading to NAD^+^ deletion and mitochondrial dysfunction; aggravating the dysfunction of Na_V_1.5 and Kir6.2 channels in cardiomyocytes, thereby inducing ischemic arrhythmias	Up	^129^
circ-JA760602	EGR1 E2F1	Inhibiting the nuclear translocation of EGR1 and E2F1, thereby suppressing the transcriptional activation of *BCL2* and promoting hypoxia-induced cardiomyocyte apoptosis	Up	^127^
circRERE	PUM2	Promoting the degradation of *Uhrf1* mRNA, which leads to reduced methylation of the *Drp1* promoter, upregulated *Drp1* expression, and consequently exacerbated mitochondrial damage.	Up	^132^
PYRCR	DRG2	Interfering with the interaction between DRG2 and DRP1, thereby reducing mitochondrial fission and pyroptosis of cardiomyocytes	Down	^133^
circDhx32	FOXO1	Inhibiting the expression of *AdipoR1*, thereby intensifying the inflammatory response of myocardial I/R injury	Up	^86^
circ-ZNF609	YTHDF3	Regulating the binding of YTHDF1 and YTHDF2 to *Yap* mRNA to control the expression of *Yap*	Up	^134^
circSamd4	VCP	Promoting the mitochondrial translocation of VCP, which subsequently binds to VDAC1 and reduces its protein expression, thereby weakening the opening of the mPTP and reducing oxidative stress.	Unknown	^84^
circFndc3b	FUS	Reducing FUS levels and promoting the upregulation of VEGF, thereby enhancing angiogenesis, limiting infarct size, and promoting cardiac repair	Down	^135^
circHipk3	Notch1	Increasing the acetylation of the Notch1 intracellular domain (N1ICD), stabilizing it, promoting its nuclear translocation, and ultimately enhancing cardiomyocyte proliferation	Unknown	^83^

It is worth noting that the same circRNA may be involved in different mechanisms in different cell types. For example, circHipk3 acts as a sponge for miR-133a in coronary artery endothelial cells to regulate connective tissue growth factor (*CTGF*) expression without interacting with the Notch1 intracellular domain (N1ICD).^83^ In cardiomyocytes, however, circHipk3 promotes proliferation by interacting with N1ICD instead of sponging miR-133a.^83^

Through existing studies, we have learned that circRNAs play a wide range of roles in IHD, including regulating apoptosis, necroptosis, autophagy, inflammation, oxidative stress, angiogenesis, fibrosis, and other processes. These functions are mediated through the regulation of relevant signaling pathways.^84^^,^^121^^,^^122^^,^^130^^,^^136^^,^^137^ Examples include the following. The GSK3β/β-catenin pathway is located downstream of the circSNRK/miR-103-3p/SNRK axis. SNRK promotes the accumulation of β-catenin by regulating the phosphorylation of GSK3β, while counteracting the increase in the cleaved-caspase-3/caspase-3 ratio, the up-regulation of BCL2-associated X protein (BAX), and the decreased expression of BCL2 apoptosis regulator (BCL-2) in cardiomyocytes. This ultimately promotes cell proliferation and reduces apoptosis.^121^ Down-regulation of circ_0060745 may inhibit cardiomyocyte apoptosis and the inflammatory response by suppressing the activation of NF-κB.^138^ Knockdown of circRNA Pum1_0014 inhibits the expression of neurofibromin 2 (*Nf2*) and reduces cardiomyocyte apoptosis by activating the vascular endothelial growth factor/p21 (RAC1) activated kinase 1 (VEGF/PAK1) pathway.^114^ Cardiac-necroptosis-associated circRNA (CNEACR) can mitigate myocardial damage caused by necroptotic cell death in ischemic heart diseases through the histone deacetylase 7/forkhead box protein A2/receptor-interacting protein kinase 3 (HDAC7/FOXA2/RIPK3) axis.^130^ The circ_ZNF512/miR-181d-5p/early growth response 1 (EGR1) axis regulates autophagy in myocardial I/R injury by up-regulating mammalian target of rapamycin complex 1 (mTORC1), which in turn down-regulates transcription factor EB (TFEB).^116^ CircFoxo3 inhibits autophagy by suppressing the lysine acetyltransferase 7/high mobility group box 1 (KAT7/HMGB1) axis, thereby alleviating MI-induced cardiac dysfunction.^136^ By sponging miR-544, circMIRIAF activates the Notch1 signaling pathway through up-regulating WD repeat domain 12 (WDR12), and thus regulates oxidative stress and inflammatory responses, consequently aggravating myocardial I/R injury.^119^ The CircSamd4/valosin containing protein/voltage-dependent anion channel 1 (CircSamd4/VCP/VDAC1) pathway plays an important role in reducing myocardial oxidative stress.^84^ CircFASTKD1 inhibits angiogenesis and increases infarct size by inhibiting the Yes-associated protein (YAP) signaling pathway.^122^ CircRNA 010567 may regulate cardiac fibrosis via the TGF-β1 signaling pathway.^137^ Although many circRNA–miRNA–mRNA networks and circRNA-interacting proteins have been identified, and progress has been made in studying their downstream signaling pathways, the specific pathways regulated by most circRNAs remain largely unknown. Additional research is required to clarify the comprehensive mechanisms by which circRNAs regulate IHD progression.

### circRNAs are involved in the regulation of HF

Multiple differentially expressed circRNAs were detected in plasma samples from HF patients compared with healthy volunteers, suggesting the potential role of circRNAs in HF.^139^ Although the regulation of HF by circRNAs has not been as extensively studied as their involvement in cardiac hypertrophy and IHD, several mechanisms have been identified.

The synthesis of circRNAs is regulated in HF. Reduced RNA editing is a characteristic of failing human hearts and may be related to the up-regulation of circRNAs.^140^ Adenosine-to-inosine (A-to-I) RNA editing is a major myocardial editing event that inhibits the formation of double-stranded RNA structures, facilitates the splicing of linear mRNA, and suppresses the formation of circRNA. This process involves adenosine deaminase acting on RNA 2 (ADAR2), which has been found to be reduced in failing hearts. The decreased expression of *ADAR2* increases the levels of circRNAs, including the formation of circAKAP13. Subsequently, excess circAKAP13 impairs the sarcomere regularity of cardiomyocytes, contributing to the development of HF.^140^

The most intensively studied mechanism in HF is the circRNA–miRNA–mRNA network. In Table 6, we have listed the identified circRNA–miRNA–mRNA interactions, including one down-regulated circRNA (circSnx12) and five up-regulated circRNAs (circRNA_0030042, circ_0091761, circSnap47, circ-HIPK3, and circ_0040414) in HF. Among them, a pressure overload-induced HF model was used to study circSnx12 and circRNA_0030042, while an ischemic HF model was used to study circ_0091761, circSnap47, and circ-HIPK3. These circRNAs contribute to a wide range of biological activities, such as iron metabolism, apoptosis, cell proliferation, and inflammatory response, thereby regulating the progression of HF.^141^, ^142^, ^143^, ^144^, ^145^, ^146^ Apart from their miRNA-sponging role, circRNAs potentially control miRNA expression by modulating the methylation of miRNA genes. For example, autophagy-related circular RNA (ACR) can increase the methylation of the miR-532 gene promoter, thereby decreasing its expression.^147^ This mechanism is related to the apoptosis of cardiomyocytes during hypoxia. Under hypoxic conditions, overexpression of ACR reduces apoptosis, while miR-532 aggravates apoptosis. In patients with chronic heart failure of ischemic origin, ACR expression was down-regulated, which may promote apoptosis through miR-532 up-regulation.^147^ In addition to regulating miRNA expression, microproteins encoded by circRNAs have been implicated in the progression of HF. For instance, Cdyl2-60aa, translated from circCDYL2 in an IRES-dependent manner, binds to apoptotic protease activating factor 1 (APAF1) and inhibits its ubiquitination by heat shock protein 70 (HSP70), thereby promoting cardiomyocyte apoptosis. This mechanism will facilitate the transition from MI to HF.^148^

**Table 6:** Identified circRNA–miRNA–mRNA networks in heart failure.

circRNA	miRNA	mRNA	Function	Expression of circRNA in heart failure	Reference
circSnx12	miR-224-5p	*Fth1*	Inhibiting ferroptosis	Down	^141^
circRNA_0030042	miR-568	*PRG4*	Promoting apoptosis and ferroptosis	Up	^146^
circ_0091761	miR-335-3p	*Acsl4*	Promoting ferroptosis	Up	^142^
circSnap47	miR-223-3p	Unknown; possibly a component of the MAPK signaling pathway	Promoting apoptosis and inflammatory response	Up	^143^
circ-HIPK3	miR-17-3p	*Adcy6*	An adrenaline helper with the potential to promote heart failure	Up	^145^
circ_0040414	miR-186-5p	*PTEN*	Inhibiting AKT signaling activity and cell proliferation, and promoting apoptosis and inflammatory response	Up	^144^

Growing evidence has illuminated circRNA functions in cardiac pathogenesis, revealing multiple regulatory networks. Firstly, the biogenesis of circRNAs is tightly regulated, encompassing both the transcription of their mRNA precursors and the subsequent cyclization process, while their stability is also subject to precise control. Subsequently, these differentially expressed circRNAs further modulate downstream signaling pathways primarily through circRNA–miRNA–mRNA networks and interactions with proteins (Fig. 4B and C). Other regulatory mechanisms have also been explored, including gene methylation regulation, miRNA maturation promotion, and microprotein coding. As numerous mechanisms are revealed, we anticipate that circular RNAs will offer novel opportunities for heart disease intervention.

## The medical value of ncRNAs: Diagnosis and treatment

### ncRNAs can serve as diagnostic biomarkers

The circulatory system contains a large number of ncRNAs, which exist either as free molecules or are encapsulated within EVs. These circulating ncRNAs may originate from cardiac cells. Under conditions of myocardial stress or injury, cardiomyocytes enhance EV production and alter their cargo, facilitating intercellular communication.^5^^,^^17^ Additionally, the protective encapsulation by EVs enables ncRNAs to remain quantifiable in circulation.^38^ Therefore, the detection of specific ncRNAs in the circulatory system may provide valuable insights for the diagnosis and prognosis of diseases.^5^^,^^17^ Consistent with this view, a recent study further demonstrates the potential of ncRNAs as biomarkers by investigating the distribution characteristics of tsRNAs inside and outside cells under stress conditions. This study indicates that the extracellular tsRNA expression profiles of cardiomyocytes and cardiac fibroblasts differ significantly under three different stress conditions: nutritional deprivation, hypoxia, and oxidative stress. These expression characteristics can distinguish samples exposed to different stressors, and their discriminatory power is significantly better than that of miRNA. More importantly, this tsRNA expression profile feature has been preliminarily verified in the plasma of patients undergoing cardiac surgery with cardiopulmonary bypass.^41^ Taken together, these findings lend strong support to the promise of ncRNAs in medical applications.

Currently, transcriptomic data have revealed a variety of ncRNA species that are differentially expressed in cardiac hypertrophy, IHD, and HF, and have demonstrated diagnostic value. Examples are as follows: Compared with the healthy group, piR-hsa-9010, piR-hsa-28646, and piR-hsa-23619 were significantly up-regulated in the serum of acute myocardial infarction patients but not in the HF or coronary heart disease groups. This indicates that these piRNAs are specific diagnostic markers for acute myocardial infarction. Moreover, receiver operating characteristic curve analysis has shown that they possess high diagnostic value.^36^ Based on the area under the receiver operating characteristic curve analysis, Xu et al suggested that tRF-21-SWRYVMMV0 and tRF-21-NB8PLML3E could emerge as optimal diagnostic candidates for pathological cardiac hypertrophy, demonstrating persistent diagnostic accuracy from early to advanced stages.^49^ CircDNAJC6, circTMEM56, and circMBOAT2 can distinguish patients with hypertrophic cardiomyopathy from healthy volunteers. Furthermore, circTMEM56 and circDNAJC6 can function as molecular gauges of hypertrophic obstructive cardiomyopathy progression.^149^ Hsa_circ_0062960 has a value of 0.838 for the area under the receiver operating characteristic curve, supporting its candidacy as a circulating diagnostic for HF.^139^ Lower ACR levels are associated with higher mortality in chronic heart failure patients, suggesting that ACR can serve as a prognostic factor.^147^ Additionally, multiple circRNAs exhibit biomarker characteristics for MI, including circPRDM5, circSLC8A1, cZNF292, and circ_PPARA.^81^^,^^82^^,^^150^^,^^151^ Among these, combining serum circPRDM5 with cardiac troponin T (cTnT) and creatine kinase-MB (CK-MB) can enhance the sensitivity of acute myocardial infarction diagnosis.^150^ Similarly, cZNF292 combined with clinical data can effectively predict acute myocardial infarction.^151^

Detecting ncRNA markers also requires technological advancements. The AlkB-facilitated RNA Methylation sequencing (ARM-Seq) developed by Cozen et al significantly enhanced the sensitivity of tsRNA detection by pretreating RNA samples with the AlkB enzyme before the reverse transcription step to remove methylation modifications.^41^^,^^152^ Broto's team engineered the CrisprZyme diagnostic platform, where Pt@Au nanozymes exhibit high catalytic activity, enabling amplification-free, quantitative RNA detection in complex samples, including ncRNA species. circRNA has been shown to be detectable by CrisprZyme. Moreover, CrisprZyme can identify differentially expressed ncRNAs in blood samples from MI patients. Therefore, the detection of ncRNA markers using this technique is expected to be applied to the diagnosis of heart disease in the future.^153^

### ncRNAs represent promising therapeutic targets for heart diseases

Current treatments for cardiovascular diseases often overlook individual differences among patients. RNA therapy, however, offers new hope for addressing this limitation.^154^ Commonly used ncRNA-targeted therapies primarily involve two approaches: supplementing inhibited RNA through RNA mimics or blocking over-activated RNA through RNA inhibitors.^9^^,^^154^ Frequently employed delivery systems include viral vectors and lipid nanoparticles.^5^ At present, research on targeting ncRNAs mainly focuses on miRNAs. The therapeutic potential of other ncRNA species remains largely unexplored. However, animal studies have shown promising therapeutic effects.^13^ For example, mice treated with CHAPIR antagomir exhibited reduced hypertrophic responses, lessened fibrotic burden, and enhanced cardiac performance compared with controls.^32^ Similarly, CFRPi antagomir treatment significantly alleviated fibrotic lesions and improved cardiac function in transverse aortic constriction mice. Moreover, *in vivo* CFRPi knockout suggests a more stable and convenient method for regulating *Apln* expression, potentially addressing APLN-related clinical challenges.^30^ Tsr007330 agomir injection into the MI rat heart tissue improved cardiac function and increased myocardial survival.^51^ Artificial circRNA sponges (circmiRs) designed to target miR-132 and miR-212 reduced the characteristics of cardiac hypertrophy in transverse aortic constriction mice.^155^ Silencing circITGa9 by delivering small interfering RNAs (siRNAs) or blocking circITGa9 binding to tropomyosin 3 (TPM3) with oligonucleotides reduced fibrosis and improved cardiac function.^68^ Nevertheless, the clinical effectiveness of these treatments may require further evaluation. It should not be overlooked that RNA therapy still faces challenges, such as off-target effects and the delivery of RNA drugs to the designated site of action. In addition, the toxicity and long-term safety of RNA drugs require further evaluation.^154^ Therefore, RNA therapy still has a long way to go, and more clinical trials and data must be conducted to validate its therapeutic potential and clinical safety.

ncRNAs could also serve as regulatory targets for small-molecule drugs or herbal extracts. In MI, the circPAN3/miR-221/PTEN axis regulates apoptosis. Quercetin demonstrates cardioprotective effects by down-regulating circPAN3 expression, which subsequently elevates miR-221-mediated inhibition of *Pten*, thereby decreasing cleaved caspase-3 levels and infarct size.^89^ The up-regulation of circ003593 in myocardial I/R injury can be attenuated by *Malva sylvestris* L. extract pretreatment, which exerts its protective effect through this mechanism.^156^ In heart failure with preserved ejection fraction, statins improve endothelial function through a molecular mechanism involving activator protein 2 alpha (AP-2α)-mediated up-regulation of circRNA-RBCK1. This circRNA interacts with miR-133a in cardiac endothelial cells to inhibit its activity, thereby improving diastolic function through the GTP cyclohydrolase 1/tetrahydrobiopterin/endothelial NO synthase (GTPCH1/BH4/eNOS) signaling pathway and preventing heart failure with preserved ejection fraction.^157^ The anti-fibrotic effects of bufalin and lycorine may be related to increased levels of cerebellar degeneration-related protein 1 antisense RNA (CDR1as).^158^ Therefore, understanding the mechanisms of ncRNAs and screening for targeted drugs may offer promising prospects for the treatment of heart disease.

## Conclusion and future perspectives

Heart disease remains one of the major threats to human health and life worldwide. Although progress has been made in medicine, the associated risks continue to pose a serious challenge. Moreover, current treatment methods are not sufficiently effective in addressing the differences between individual patients, making it critically important to explore new and suitable targeted approaches. The regulatory role of ncRNAs in heart disease positions them as potential candidate targets. In this paper, we summarize the research progress of snoRNAs, piRNAs, tsRNAs, and circRNAs in cardiac hypertrophy, IHD, and HF. These ncRNAs are differentially expressed in all three heart diseases and demonstrate significant potential as diagnostic markers, suggesting their important regulatory functions and promise for medical applications. Mechanistically, important advances have been made in understanding the downstream effects of circRNAs. Existing studies have mainly revealed two possible mechanisms of action: through the circRNA–miRNA–mRNA network and/or through interactions with proteins. Additionally, the microprotein-coding potential of certain circRNAs may represent an important future research direction in this field. The research on the downstream mechanism of piRNAs is also gradually emerging. The most well-known mechanisms include directly targeting mRNA for gene silencing and regulating epigenetic modification protein activity. tsRNAs have also been found to regulate heart disease development through two distinct mechanisms: mediating gene silencing via base pairing and modulating gene expression through protein interactions. In contrast, little is known about the mechanism by which snoRNAs are involved in the regulation of heart disease. However, given the known functions of snoRNAs, we speculate that they may be involved in regulation through epigenetic modifications, protein interactions, or other potentially non-canonical functions, although further experimental data are needed to confirm this. The discovery of these mechanisms provides a theoretical foundation for targeting snoRNAs, piRNAs, tsRNAs, and circRNAs. Although clinical data are not yet available, therapies targeting these ncRNAs have shown progress and promising therapeutic effects in animal models.

Nevertheless, many unresolved issues remain. Firstly, the regulation of biogenesis and degradation of these ncRNAs in heart disease, as well as their impact on downstream pathways, is not fully understood. Specifically, among the four discussed ncRNAs, especially the research on snoRNAs, piRNAs, and tsRNAs in the context of heart disease currently leans more toward reporting associations rather than explaining the causative mechanisms behind these changes or their functional consequences. Therefore, further studies are needed to provide a comprehensive understanding of these processes. A critical problem that needs to be solved is whether the observed alterations in these ncRNAs represent a direct cause of the initiation and progression of heart disease, a contributing participant in the pathological processes, or merely a secondary consequence of the disease. Secondly, interactions between different ncRNA species exist, and it would be valuable to investigate how these interactions collectively regulate heart disease. Thirdly, beyond the ncRNA species discussed in this paper (and the well-established roles of miRNAs and lncRNAs), whether other, less-characterized ncRNAs also play regulatory roles in heart disease remains to be explored. Fourthly, more clinical studies are required to evaluate the diagnostic and therapeutic potential of such ncRNAs. Finally, the methods and safety of RNA-targeted therapies need further evaluation, as current RNA-based treatments still exhibit certain side effects. A limitation of this paper is its exclusive focus on the roles of snoRNAs, piRNAs, tsRNAs, and circRNAs in cardiac hypertrophy, IHD, and HF. There are numerous other types of heart diseases, such as myocarditis, which are not addressed in this article. Understanding the mechanisms of these ncRNAs across various types of heart disease will facilitate the development of targeted treatments.

Overall, snoRNAs, piRNAs, tsRNAs, and circRNAs have emerged as significant players in the field of heart disease. They provide new insights into the pathogenesis of heart disease and hold promise as potential targets for diagnosis and treatment. Therefore, it is essential to intensify research on these ncRNAs in the future.

## CRediT authorship contribution statement

**Jingyi Wang:** Writing – original draft. **Jingyi Xie:** Writing – review & editing, Data curation. **Hao Chen:** Writing – review & editing. **Wenxu Wang:** Writing – review & editing. **Qing Liu:** Writing – review & editing, Funding acquisition. **Xuanzhen Guo:** Writing – review & editing, Funding acquisition. **Kun Wang:** Writing – review & editing. **Jie Ju:** Writing – review & editing, Funding acquisition.

## Funding

This work was supported by the National Natural Science Foundation (China) (No. 82500356), the Shandong Provincial Natural Science Foundation (No. ZR2025QC863), the PhD research startup foundation of Shandong Second Medical University (China) (No. 0215500104), the Weifang science and technology development plan project for medicine (China) (No. 2025YX039, 2024YX036), and the Shandong Second Medical University innovation and entrepreneurship training program (China) (No. X2024281).

## Conflict of interests

The authors declared no competing interests.
